# 2,4-Dichloro-*N*-*p*-tolyl­benzamide

**DOI:** 10.1107/S1600536809034710

**Published:** 2009-09-26

**Authors:** Aamer Saeed, Rasheed Ahmad Khera, Hummera Rafique, Jim Simpson, Roderick G. Stanley

**Affiliations:** a Department of Chemistry, Quaid-i-Azam University, Islamabad 45320, Pakistan; b Department of Chemistry, University of Otago, PO Box 56, Dunedin, New Zealand

## Abstract

In the title compound, C_14_H_11_Cl_2_NO, the C—N—C(=O)—C amide unit is almost planar (r.m.s. deviation = 0.0317 Å) and subtends dihedral angles of 65.93 (6) and 29.45 (7)°, respectively, to the dichloro­benzene and tolyl rings. The two aromatic rings are inclined at 37.92 (6)° to one another. In the crystal structure, N—H⋯O hydrogen bonds link the mol­ecules into chains along *b*. Additional weak C—H⋯Cl and C—H⋯O hydrogen bonds combine with C—H⋯π and very weak π–π contacts [*Cg*⋯*Cg* distance = 4.0217 (12) Å] to stack the mol­ecules down *b*.

## Related literature

For background to our work on benzamide derivatives, see: Saeed *et al.* (2008 ▶). For related structures see: Zhou & Zheng (2007 ▶); Gowda *et al.* (2008*a* ▶,*b* ▶,*c* ▶, 2009 ▶); Chopra & Guru Row (2005 ▶).
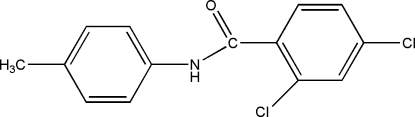


## Experimental

### 

#### Crystal data

C_14_H_11_Cl_2_NO
*M* *_r_* = 280.14
Monoclinic, 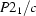

*a* = 9.0884 (18) Å
*b* = 9.825 (2) Å
*c* = 14.167 (3) Å
β = 94.208 (9)°
*V* = 1261.6 (4) Å^3^
*Z* = 4
Mo *K*α radiation
μ = 0.50 mm^−1^
*T* = 89 K
0.33 × 0.26 × 0.06 mm

#### Data collection

Bruker APEXII CCD diffractometer
Absorption correction: multi-scan (*SADABS*; Bruker, 2006 ▶) *T*_min_ = 0.753, *T*_max_ = 0.970
20982 measured reflections
4465 independent reflections
3463 reflections with *I* > 2σ(*I*)
*R*_int_ = 0.044

#### Refinement

*R*[*F* ^2^ > 2σ(*F* ^2^)] = 0.041
*wR*(*F* ^2^) = 0.132
*S* = 1.15
4465 reflections
167 parameters
H atoms treated by a mixture of independent and constrained refinement
Δρ_max_ = 0.57 e Å^−3^
Δρ_min_ = −0.49 e Å^−3^

### 

Data collection: *APEX2* (Bruker, 2006 ▶); cell refinement: *APEX2* and *SAINT* (Bruker, 2006 ▶); data reduction: *SAINT*; program(s) used to solve structure: *SHELXS97* (Sheldrick, 2008 ▶); program(s) used to refine structure: *SHELXL97* (Sheldrick, 2008 ▶) and *TITAN2000* (Hunter & Simpson, 1999 ▶); molecular graphics: *SHELXTL* (Sheldrick, 2008 ▶) and *Mercury* (Macrae *et al.*, 2006 ▶); software used to prepare material for publication: *SHELXL97*, *enCIFer* (Allen *et al.*, 2004 ▶), *PLATON* (Spek, 2009 ▶) and *publCIF* (Westrip, 2009 ▶).

## Supplementary Material

Crystal structure: contains datablocks global, I. DOI: 10.1107/S1600536809034710/fl2252sup1.cif

Structure factors: contains datablocks I. DOI: 10.1107/S1600536809034710/fl2252Isup2.hkl

Additional supplementary materials:  crystallographic information; 3D view; checkCIF report

## Figures and Tables

**Table 1:** Hydrogen-bond geometry (Å, °)

*D*—H⋯*A*	*D*—H	H⋯*A*	*D*⋯*A*	*D*—H⋯*A*
N1—H1*N*⋯O1^i^	0.86 (2)	2.14 (2)	2.9867 (17)	168 (2)
C12—H12⋯Cl1^ii^	0.95	2.91	3.7372 (17)	146
C6—H6⋯O1^iii^	0.95	2.67	3.619 (2)	175
C7—H7⋯*Cg*2^iv^	0.95	2.65	3.4865 (17)	147

Symmetry codes: (i) 
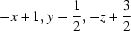
; (ii) 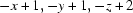; (iii) 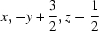; (iv) 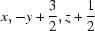. *Cg*2 is the centroid of the C8–C13 ring.

**Fig. 1.**
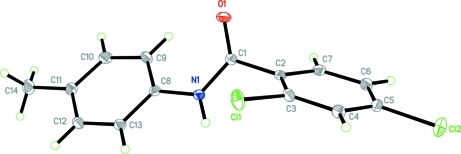
The structure of (I) with displacement ellipsoids for the non-hydrogen atoms drawn at the 50% probability level.

**Fig. 2.**
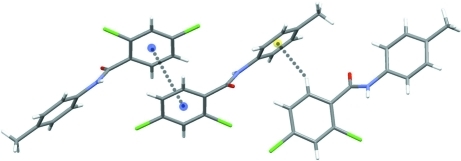
π–π and C—H···π interactions in (I). Contacts are shown as dotted lines,the coloured spheres represent the ring centroids.

**Fig. 3.**
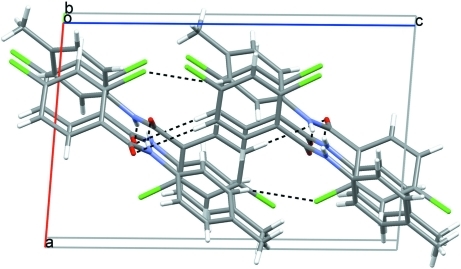
Crystal packing of (I) viewed down the b axis, with hydrogen bonds drawn as dashed lines.

## supplementary crystallographic information

### Comment

The background to our work on benzamide derivatives has been described in a previous paper (Saeed *et al.*, 2008). In the title compound (I), Fig. 1, the C8–N1–C1(O1)–C2 amide unit is planar, r.m.s. deviation 0.0317 Å, and subtends dihedral angles of 65.93 (6)° and 29.45 (7)° respectively to the C2···C7 dichlorobenzene and C8···C13 tolyl rings. The two aromatic rings are inclined at 37.92 (6)° to one another. Bond distances within the molecule are normal and similar to those observed in comparable structures (Zhou & Zheng, 2007; Gowda *et al.* 2008*a*,b,c 2009; Chopra & Guru Row, 2005).

In the crystal structure N—H···O hydrogen bonds link molecules in a head to tail fashion into rows along *b*. C7—H7···π and weak, inversion related π–π contacts involving adjacent dichlorobenene rings [*Cg*···*Cg* distance 4.0217 (12), symmetry operation 1 - *x*, 1 - *y*, 1 - *z*] are also observed, Table 1, Fig 2. These together with additional C—H···Cl and C—H···O hydrogen bonds link the stacks of molecules alternately head to head and head to tail down the *b* axis, Fig. 3.

### Experimental

2,4-Dichlorobenzoyl chloride (5.4 mmol) in CHCl_3_ was treated with *p*-toluidine(21.6 mmol) under a nitrogen atmosphere at reflux for 3 h. Upon cooling, the reaction mixture was diluted with CHCl_3_ and washed consecutively with aq 1 *M* HCl and saturated aq NaHCO_3_. The organic layer was dried over anhydrous magnesium sulfate and concentrated under reduced pressure. Crystallization of the residue by evaporation from CHCl_3_ afforded the title compound (84%) as colourless needles: Anal. calcd. for C_14_H_11_Cl_2_NO,: C, 60.02; H, 3.96; N, 5.00%; found: C, 60.06; H, 3.92; N, 5.10%

### Refinement

The H atom bound to N1 was located in a difference Fourier map and its coordinates were refined with *U*_iso_=1.2*U*_eq_ (N). All other H-atoms were placed in calculated positions and refined using a riding model with d(C—H) = 0.95 Å, *U*_iso_=1.2*U*_eq_ (C) for aromatic and 0.98 Å, *U*_iso_ = 1.5*U*_eq_ (C) for CH_3_ H atoms.

### Crystal data

**Table:**

C_14_H_11_Cl_2_NO	*F*(000) = 576
*M**_r_* = 280.14	*D*_x_ = 1.475 Mg m^−^^3^
Monoclinic, *P*2_1_/*c*	Mo *K*α radiation, λ = 0.71073 Å
Hall symbol: -P 2ybc	Cell parameters from 5859 reflections
*a* = 9.0884 (18) Å	θ = 2.5–33.0°
*b* = 9.825 (2) Å	µ = 0.50 mm^−^^1^
*c* = 14.167 (3) Å	*T* = 89 K
β = 94.208 (9)°	Irregular fragment, colourless
*V* = 1261.6 (4) Å^3^	0.33 × 0.26 × 0.06 mm
*Z* = 4	

### Data collection

**Table:**

Bruker APEXII CCD diffractometer	4465 independent reflections
Radiation source: fine-focus sealed tube	3463 reflections with *I* > 2σ(*I*)
graphite	*R*_int_ = 0.044
ω scans	θ_max_ = 33.1°, θ_min_ = 2.9°
Absorption correction: multi-scan (*SADABS*; Bruker, 2006)	*h* = −13→13
*T*_min_ = 0.753, *T*_max_ = 0.970	*k* = −14→13
20982 measured reflections	*l* = −21→21

### Refinement

**Table:**

Refinement on *F*^2^	Primary atom site location: structure-invariant direct methods
Least-squares matrix: full	Secondary atom site location: difference Fourier map
*R*[*F*^2^ > 2σ(*F*^2^)] = 0.041	Hydrogen site location: inferred from neighbouring sites
*wR*(*F*^2^) = 0.132	H atoms treated by a mixture of independent and constrained refinement
*S* = 1.15	*w* = 1/[σ^2^(*F*_o_^2^) + (0.0688*P*)^2^ + 0.2064*P*] where *P* = (*F*_o_^2^ + 2*F*_c_^2^)/3
4465 reflections	(Δ/σ)_max_ = 0.001
167 parameters	Δρ_max_ = 0.57 e Å^−^^3^
0 restraints	Δρ_min_ = −0.48 e Å^−^^3^

### Special details

**Table:**

Geometry. All e.s.d.'s (except the e.s.d. in the dihedral angle between two l.s. planes) are estimated using the full covariance matrix. The cell e.s.d.'s are taken into account individually in the estimation of e.s.d.'s in distances, angles and torsion angles; correlations between e.s.d.'s in cell parameters are only used when they are defined by crystal symmetry. An approximate (isotropic) treatment of cell e.s.d.'s is used for estimating e.s.d.'s involving l.s. planes.
Refinement. Refinement of *F*^2^ against ALL reflections. The weighted *R*-factor *wR* and goodness of fit *S* are based on *F*^2^, conventional *R*-factors *R* are based on *F*, with *F* set to zero for negative *F*^2^. The threshold expression of *F*^2^ > σ(*F*^2^) is used only for calculating *R*-factors(gt) *etc*. and is not relevant to the choice of reflections for refinement. *R*-factors based on *F*^2^ are statistically about twice as large as those based on *F*, and *R*- factors based on ALL data will be even larger.

### Fractional atomic coordinates and isotropic or equivalent isotropic displacement parameters (Å^2^)

**Table:**

	*x*	*y*	*z*	*U*_iso_*/*U*_eq_	
C1	0.52742 (16)	0.71474 (14)	0.71812 (10)	0.0111 (3)	
C2	0.43510 (16)	0.67111 (13)	0.63071 (10)	0.0105 (3)	
C3	0.29263 (16)	0.61878 (14)	0.63334 (10)	0.0121 (3)	
C4	0.21096 (16)	0.57806 (14)	0.55106 (10)	0.0126 (3)	
H4	0.1147	0.5414	0.5539	0.015*	
C5	0.27317 (16)	0.59219 (14)	0.46479 (10)	0.0125 (3)	
C6	0.41328 (16)	0.64737 (14)	0.45883 (10)	0.0130 (3)	
H6	0.4536	0.6584	0.3993	0.016*	
C7	0.49281 (16)	0.68595 (14)	0.54224 (11)	0.0125 (3)	
H7	0.5887	0.7233	0.5391	0.015*	
C8	0.66786 (16)	0.62192 (14)	0.86070 (10)	0.0109 (3)	
C9	0.77909 (17)	0.71922 (15)	0.87165 (11)	0.0141 (3)	
H9	0.7902	0.7855	0.8239	0.017*	
C10	0.87396 (17)	0.71820 (15)	0.95361 (11)	0.0153 (3)	
H10	0.9498	0.7847	0.9607	0.018*	
C11	0.86130 (16)	0.62256 (14)	1.02572 (11)	0.0131 (3)	
C12	0.74965 (17)	0.52520 (15)	1.01274 (11)	0.0142 (3)	
H12	0.7388	0.4585	1.0603	0.017*	
C13	0.65393 (16)	0.52412 (14)	0.93137 (10)	0.0123 (3)	
H13	0.5789	0.4568	0.9238	0.015*	
C14	0.96308 (18)	0.62590 (17)	1.11523 (11)	0.0184 (3)	
H14A	0.9120	0.6683	1.1662	0.028*	
H14B	1.0515	0.6788	1.1040	0.028*	
H14C	0.9916	0.5328	1.1334	0.028*	
N1	0.56897 (14)	0.61267 (12)	0.77821 (9)	0.0120 (2)	
O1	0.56444 (13)	0.83472 (10)	0.72985 (8)	0.0168 (2)	
Cl1	0.20933 (4)	0.60815 (4)	0.73967 (3)	0.01815 (11)	
Cl2	0.17246 (4)	0.53821 (4)	0.36221 (3)	0.01910 (11)	
H1N	0.530 (2)	0.534 (2)	0.7672 (16)	0.023*	

### Atomic displacement parameters (Å^2^)

**Table:**

	*U*^11^	*U*^22^	*U*^33^	*U*^12^	*U*^13^	*U*^23^
C1	0.0116 (6)	0.0104 (6)	0.0112 (6)	0.0005 (5)	0.0004 (5)	0.0007 (5)
C2	0.0122 (6)	0.0073 (6)	0.0117 (6)	0.0008 (4)	−0.0010 (5)	0.0009 (4)
C3	0.0135 (6)	0.0120 (6)	0.0107 (6)	0.0020 (5)	0.0011 (5)	0.0023 (5)
C4	0.0108 (6)	0.0127 (6)	0.0141 (7)	−0.0008 (5)	−0.0005 (5)	0.0009 (5)
C5	0.0145 (6)	0.0111 (6)	0.0115 (6)	0.0015 (5)	−0.0020 (5)	−0.0012 (5)
C6	0.0154 (6)	0.0114 (6)	0.0123 (6)	0.0003 (5)	0.0022 (5)	0.0005 (5)
C7	0.0127 (6)	0.0095 (6)	0.0153 (7)	−0.0011 (5)	0.0005 (5)	0.0005 (5)
C8	0.0121 (6)	0.0091 (6)	0.0112 (6)	0.0016 (4)	−0.0006 (5)	−0.0013 (4)
C9	0.0154 (6)	0.0127 (6)	0.0137 (6)	−0.0026 (5)	−0.0014 (5)	0.0035 (5)
C10	0.0141 (7)	0.0134 (6)	0.0179 (7)	−0.0031 (5)	−0.0015 (6)	0.0003 (5)
C11	0.0117 (6)	0.0141 (6)	0.0133 (6)	0.0011 (5)	−0.0008 (5)	−0.0002 (5)
C12	0.0166 (7)	0.0131 (6)	0.0125 (6)	−0.0014 (5)	−0.0008 (5)	0.0026 (5)
C13	0.0130 (6)	0.0103 (6)	0.0134 (6)	−0.0019 (5)	−0.0001 (5)	0.0005 (5)
C14	0.0182 (7)	0.0217 (7)	0.0145 (7)	−0.0031 (6)	−0.0037 (6)	0.0012 (6)
N1	0.0154 (6)	0.0084 (5)	0.0115 (6)	−0.0014 (4)	−0.0034 (5)	0.0006 (4)
O1	0.0210 (6)	0.0084 (5)	0.0199 (5)	−0.0009 (4)	−0.0056 (4)	0.0009 (4)
Cl1	0.01473 (18)	0.0284 (2)	0.01157 (18)	0.00040 (13)	0.00261 (13)	0.00371 (13)
Cl2	0.01772 (19)	0.0254 (2)	0.01362 (18)	−0.00149 (13)	−0.00263 (14)	−0.00494 (13)

### Geometric parameters (Å, °)

**Table:**

C1—O1	1.2338 (17)	C8—C13	1.400 (2)
C1—N1	1.3512 (18)	C8—N1	1.4240 (18)
C1—C2	1.5062 (19)	C9—C10	1.395 (2)
C2—C3	1.396 (2)	C9—H9	0.9500
C2—C7	1.401 (2)	C10—C11	1.399 (2)
C3—C4	1.394 (2)	C10—H10	0.9500
C3—Cl1	1.7379 (16)	C11—C12	1.397 (2)
C4—C5	1.391 (2)	C11—C14	1.514 (2)
C4—H4	0.9500	C12—C13	1.392 (2)
C5—C6	1.392 (2)	C12—H12	0.9500
C5—Cl2	1.7423 (15)	C13—H13	0.9500
C6—C7	1.392 (2)	C14—H14A	0.9800
C6—H6	0.9500	C14—H14B	0.9800
C7—H7	0.9500	C14—H14C	0.9800
C8—C9	1.392 (2)	N1—H1N	0.86 (2)
			
O1—C1—N1	124.29 (13)	C8—C9—C10	119.17 (13)
O1—C1—C2	120.84 (12)	C8—C9—H9	120.4
N1—C1—C2	114.82 (12)	C10—C9—H9	120.4
C3—C2—C7	118.10 (13)	C9—C10—C11	122.27 (14)
C3—C2—C1	122.99 (13)	C9—C10—H10	118.9
C7—C2—C1	118.90 (13)	C11—C10—H10	118.9
C4—C3—C2	121.42 (14)	C12—C11—C10	117.50 (13)
C4—C3—Cl1	117.94 (12)	C12—C11—C14	121.22 (14)
C2—C3—Cl1	120.61 (11)	C10—C11—C14	121.27 (13)
C5—C4—C3	118.71 (14)	C13—C12—C11	121.19 (14)
C5—C4—H4	120.6	C13—C12—H12	119.4
C3—C4—H4	120.6	C11—C12—H12	119.4
C4—C5—C6	121.66 (13)	C12—C13—C8	120.20 (13)
C4—C5—Cl2	118.69 (11)	C12—C13—H13	119.9
C6—C5—Cl2	119.65 (12)	C8—C13—H13	119.9
C7—C6—C5	118.36 (14)	C11—C14—H14A	109.5
C7—C6—H6	120.8	C11—C14—H14B	109.5
C5—C6—H6	120.8	H14A—C14—H14B	109.5
C6—C7—C2	121.72 (13)	C11—C14—H14C	109.5
C6—C7—H7	119.1	H14A—C14—H14C	109.5
C2—C7—H7	119.1	H14B—C14—H14C	109.5
C9—C8—C13	119.66 (13)	C1—N1—C8	126.87 (12)
C9—C8—N1	123.01 (13)	C1—N1—H1N	117.5 (14)
C13—C8—N1	117.28 (12)	C8—N1—H1N	115.6 (14)
			
O1—C1—C2—C3	−115.79 (17)	C1—C2—C7—C6	179.87 (12)
N1—C1—C2—C3	66.64 (18)	C13—C8—C9—C10	−0.6 (2)
O1—C1—C2—C7	62.85 (19)	N1—C8—C9—C10	−178.01 (14)
N1—C1—C2—C7	−114.72 (15)	C8—C9—C10—C11	−0.1 (2)
C7—C2—C3—C4	2.0 (2)	C9—C10—C11—C12	0.6 (2)
C1—C2—C3—C4	−179.31 (13)	C9—C10—C11—C14	−178.33 (15)
C7—C2—C3—Cl1	−175.69 (10)	C10—C11—C12—C13	−0.5 (2)
C1—C2—C3—Cl1	2.96 (19)	C14—C11—C12—C13	178.50 (14)
C2—C3—C4—C5	−0.9 (2)	C11—C12—C13—C8	−0.2 (2)
Cl1—C3—C4—C5	176.86 (11)	C9—C8—C13—C12	0.8 (2)
C3—C4—C5—C6	−0.9 (2)	N1—C8—C13—C12	178.32 (13)
C3—C4—C5—Cl2	178.77 (11)	O1—C1—N1—C8	−4.3 (2)
C4—C5—C6—C7	1.5 (2)	C2—C1—N1—C8	173.21 (13)
Cl2—C5—C6—C7	−178.17 (11)	C9—C8—N1—C1	−26.7 (2)
C5—C6—C7—C2	−0.3 (2)	C13—C8—N1—C1	155.92 (15)
C3—C2—C7—C6	−1.4 (2)		

### Hydrogen-bond geometry (Å, °)

**Table:**

*D*—H···*A*	*D*—H	H···*A*	*D*···*A*	*D*—H···*A*
N1—H1N···O1^i^	0.86 (2)	2.14 (2)	2.9867 (17)	168 (2)
C12—H12···Cl1^ii^	0.95	2.91	3.7372 (17)	146
C6—H6···O1^iii^	0.95	2.67	3.619 (2)	175
C7—H7···Cg2^iv^	0.95	2.65	3.4865 (17)	147

Symmetry codes: (i) −*x*+1, *y*−1/2, −*z*+3/2; (ii) −*x*+1, −*y*+1, −*z*+2; (iii) *x*, −*y*+3/2, *z*−1/2; (iv) *x*, −*y*+3/2, *z*+1/2.
